# Hepatocyte polyploidization and its association with pathophysiological processes

**DOI:** 10.1038/cddis.2017.167

**Published:** 2017-05-18

**Authors:** Min-Jun Wang, Fei Chen, Joseph T Y Lau, Yi-Ping Hu

**Affiliations:** 1 Department of Cell Biology, Center for Stem Cell and Medicine, Second Military Medical University, Shanghai 200433, China; 2 Department of Molecular and Cellular Biology, Roswell Park Cancer Institute, Buffalo, NY 14263, USA

## Abstract

A characteristic cellular feature of the mammalian liver is the progressive polyploidization of the hepatocytes, where individual cells acquire more than two sets of chromosomes. Polyploidization results from cytokinesis failure that takes place progressively during the course of postnatal development. The proportion of polyploidy also increases with the aging process or with cellular stress such as surgical resection, toxic stimulation, metabolic overload, or oxidative damage, to involve as much as 90% of the hepatocytes in mice and 40% in humans. Hepatocyte polyploidization is generally considered an indicator of terminal differentiation and cellular senescence, and related to the dysfunction of insulin and p53/p21 signaling pathways. Interestingly, the high prevalence of hepatocyte polyploidization in the aged mouse liver can be reversed when the senescent hepatocytes are serially transplanted into young mouse livers. Here we review the current knowledge on the mechanism of hepatocytes polyploidization during postnatal growth, aging, and liver diseases. The biologic significance of polyploidization in senescent reversal, within the context of new ways to think of liver aging and liver diseases is considered.

## Facts

- The hepatocyte is a main contributor to liver functions, including in metabolic homeostasis, synthesis, storage, distribution, and detoxification of xenobiotic compounds.
- Hepatocyte polyploidization is a characteristic feature of mature mammalian hepatocytes and an indicator of hepatocyte senescence.
- Waves of hepatocyte polyploidization occur successively during postnatal development and again upon advanced ageing.
- The frequency and extent of hepatic polyploidy are increased in liver injury by partial hepatectomy, radiation or oxidative stress, but are reduced in hepatocellular carcinoma.
- The senescent mouse hepatocyte is rejuvenated and exhibits polyploidy reversal upon immersion into the microenvironment of a young liver.

## Open questions

- What is the significance of the waves of hepatocyte polyploidization in normal development and ageing?
- What are the roles of altered hepatocyte polyploidization status with different pathologic conditions?
- What is the causal relationship between polyploidy reversal and the rejuvenation of senescent hepatocytes?

The liver is the largest solid organ endowed with unique regenerative capacity in the mammalian body, and accounts for about 2% of the total body weight in humans and 5% in mice. It is an essential organ with multiple complex functions in metabolic homeostasis, synthesis, and distribution of nutrients and serum proteins, metabolism and storage of amino acids, vitamins, lipids, and carbohydrates, and detoxification of xenobiotic compounds.^1, 2^ Most of these functions are carried out primarily by hepatocytes that account for 70% of all liver cells.^3^ Hepatocytes have unique functions depending on their location within the liver lobule.^4^ The peripotal hepatocytes mediate amino acid and energy metabolism, lipid oxidation, and gluconeogenesis. In contrast, glycolysis, lipogenesis, and glutamine synthase are mediated by pericentral hepatocytes. Although hepatocytes are quiescent in the adult and turnover slowly with a mean life span of 200–300 days,^5^ they are able to proliferate rapidly in response to liver injury.

A characteristic feature of hepatocytes is polyploidy, which is an increase in the numbers of chromosome sets per cell. Liver polyploidy occurs during postnatal development as an age-dependent process, as well as during repair in response to injury from various stress stimuli. Polyploidization in the liver has been studied widely and summarized in excellent reviews.^6, 7^ Hepatocyte ploidy depends on the DNA content of each nucleus (for example, diploid, tetraploid, octoploid, and so on) plus the number of nuclei per cell.^8^ For example, polyploid hepatocytes can be tetraploid (for example, binucleate with two diploid nuclei or mononucleate with a single tetraploid nucleus), or octoploid (for example, binucleate with two tetraploid nuclei or mononucleate with a single octoploid nucleus). Enlarged cell size is the most obvious consequence of liver polyploidization. Different studies have reported that the volume of hepatocytes in human and mouse livers is approximately doubled with the doubling of DNA content.^9^ However, there is no significant difference in the volume of binucleated tetraploid (2 × 2n) and that of mononucleated tetraploid (4n) hepatocytes or between binucleated (2 × 4n) and mononucleated (8n) octoploid hepatocytes. Although polyploid hepatocytes were documented over a century ago, the physiologic significance of polyploidy status in liver homeostasis, regeneration, and disease is poorly understood. In this review, we will explore the possible mechanism underlying liver ployploidization, and discuss how polyploidization is regulated during liver physiological development, and its roles in aging, pathological processes, and senescent reversal.

## Hepatocyte Polyploidization During the Growth and Development of the Postnatal Liver

**Figure 1:**
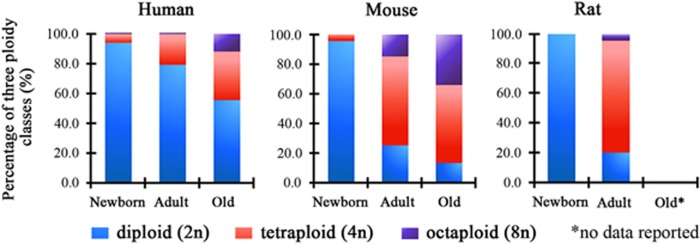
The distribution of ploidy classes in different mammals. The graphs show the percentage of diploid, tetraploid, and octoploid hepatocytes in newborn, adult, and aged human, mice and rat. Adult mice and rats were 8–12 weeks old; adult human were 20–40 years old. Aged mice were over 18 months, and aged human were over 60 years old. The data were compiled from published studies (Duncan AW, *et al.*;^13^ Wang MJ, *et al.*;^14^ Chipchase MD, *et al.*;^15^ Pandit SK, *et al.*;^16^ Margall-Ducos G, *et al.*;^17^ Gandillet A, *et al.*;^18^ Kudryavtsev BN, *et al.*;^19^ Duncan AW, *et al.*^20^)

All hepatocytes are diploid in the newborn liver. During postnatal growth, the liver parenchyma undergoes dramatic changes with gradual polyploidization, with the emergence of hepatocytes of several ploid classes.^8, 10^ Polyploidization begins from the first 3 weeks postnatal, generating binucleated tetraploid (2 × 2n) or mononucleated tetraploid (4n) hepatocytes. Octoploid (binucleated 2 × 4n and mononucleated 8n) hepatocytes begin to accumulate in significant numbers during the second and third months.^8, 11^ Hepatocyte polyploidization reaches a plateau upon animal maturity. The degree of liver polyploidization varies between mammals (Figure 1).^12, 13, 14, 15, 16, 17, 18, 19, 20^ 80–90% of hepatocytes in adult C57BL mice are polyploid,^13, 14, 15, 16^ and 70–80% of adult rat hepatocytes are polyploid.^17, 18^ In the adult human liver, the percentage of polyploidy is more than 20%.^19, 20^

**Figure 2:**
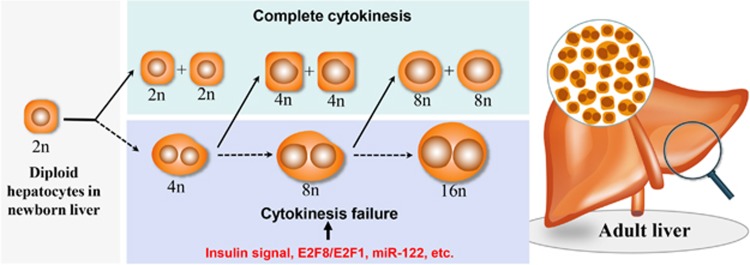
Polyploidization during postnatal liver growth. From the postnatal growth, a diploid hepatocyte can give rise to two diploid hepatocytes with successful cytokinesis or follows with cytokinesis failure and generates a tetraploid hepatocyte with two diploid nuclei. The binucleated tetraploid hepatocyte then follows a new round cell cycle, generating two mononucleated tetraploids or one binucleated octoploidy. In the adult liver parenchyma, it consists of diploid, tetraploid and octoploid hepatocytes. Insulin signaling, E2F transcription factors including E2F8 and E2F1, and miR-122 have been reported to regulate cytokinesis failure and be crucial for liver polyploidization during postnatal development

Cytokinesis failure is the main mechanism of polyploidization during postnatal liver development (Figure 2). In successful cytokinesis, a diploid hepatocyte gives rise to two diploid hepatocytes, but an incomplete cytokinesis generates one tetraploid hepatocyte with two diploid nuclei.^8, 17^ The binucleated tetraploid hepatocyte retains the capability to undergo future DNA replication with successful cyrokinesis, generating two mononucleated tetraploid cells. However, if cytokinesis fails again during mitosis of a mononucleated tetraploid cell, a binucleated octoploid hepatocyte results. In this way, progressive polyploidization with the formation of one or two nucleated tetraploid and octoploid cells appears successively in the liver parenchyma. Cytokinesis failure is reported to be regulated by insulin signaling that may act via the phosphoinositide 3-kinase (PI3K)-protein kinase B (Akt)-cytoskeleton regulation pathway.^11, 21, 22^ These studies demonstrate that reduced circulating insulin levels resulted in the decreased generation of binucleated tetraploid hepatocytes, while elevated insulin levels increased the formation of binucleated tetraploid hepatocytes. Inhibition of PI3K/Akt phosphorylation blocked these failed cytokinetic event, and complete cytokinesis occurs followed by actin cytoskeleton polarization, cytoskeleton reorganization, and RhoA recruitment.

A number of other factors in addition to the insulin-signaling pathway may contribute also to liver polyploidization (Table 1). E2F transcription factors have been implicated as crucial for liver polyploidization during postnatal development.^16, 23, 24^ E2F8 deficiency induces the expression of E2F target genes, promoting cytokinesis and preventing liver polyploidization. In contrast, E2F1 deficiency inhibits cytokinesis and enhances liver polyploidization. In mouse models, silencing of cell-cycle-regulated factors CDK1, Skp2, Ccne2, p21, p53, pRb, survivin, Ssu72, and nucleotide excision repair gene ERCC1 led to enhanced liver polyploidization, but silence of Ccne1 repressed liver polyploidy.^15, 25, 26, 27, 28, 29, 30, 31, 32, 33^ Importantly, a recent study concluded that miR-122 is not only necessary but also sufficient for hepatic polyploidization.^34^ Downregulating miR-122 expression decreases polyploid hepatocytes, and this trend is reversible by miR-122 over-expression. MiR-122 antagonizes the expression of the pro-cytokinesis effectors *Cux1, Rhoa, Mapre1, Iqgap1, Nedd4l, and Slc25a34*, leading to cytokinesis failure and expansion of binucleated hepatocytes.

## Hepatocyte Polyploidization in the Aged Liver and its Reversal Phenomenon

A second wave of high polyploidization occurs as an aging-dependent process (Figure 1).^14, 15, 19^ Most hepatocytes are diploid in young individuals,^20^ where the relative number of polyploidy cells generally does not exceed 15% in 20-year-old adult humans.^19^ However, the fraction of polyploid hepatocytes increases to 42% in the liver of an 80-year-old adult.^19^ Our previous study,^14^ corroborating this aging-related trend in the mouse, documented that the percentage of octoploid hepatocytes in mouse liver increases from 16.8±5.1% at 2 months to 34.06±1.8% at 18 months.

Ageing-related hepatocyte polyploidy has been regarded as a manifestation of hepatic cellular senescence. The liver regenerative capacity after a 70% hepatectomy is decreased in older animals with greater proportions of polyploid hepatocytes.^35, 36^ It has been shown previously that hepatocytes are formed adjacent to the portal zone and stream toward the terminal hepatic vein as they age.^37^ The DNA content of hepatocytes also increases with their age.^37, 38^ As reported in our previous study, diploid, tetraploid, and octoploid hepatocytes from 2-month mice rarely expressed senescent markers, including p16^ink4α^, p21 and p53, and have no significant difference in liver repopulation after transplanting into the recipient livers. However, the expression of p16^ink4α^, p21 and p53 in diploidy, tetraploidy, and octoploidy from the 18-month mice were higher than from the 2-month mice. Moreover, the expression levels of p16^ink4a^, p21, and p53 in the 18-month mice were significantly higher in the octoploid than in the diploid and tetraploid hepatocytes. Importantly, after transplantation, the proliferative capacity and liver repopulation of octoploid hepatocytes were lower than diploid and tetraploid donor hepatocytes.^14^ Similar to *in vivo* hepatocytes, cultured primary hepatocytes from the 18-month-old mice contained more SA-β-gal positive cells and fewer bromodeoxyuridine (BrdU)-positive cells as compared with the 2-month mice.^14^ Our study correlated increased polyploid hepatocytes in older mice with senescence, suggesting that polyploidy may induce senescence-type changes during aging and possible association with liver disease.

The p16^ink4α^ and p53-p21 pathways regulating hepatocyte senescence may be a mechanism of polyploidization during aging. The level of p16^ink4α^ was also increased in fibroblasts as they approached senescence, accompanied with formation of polyploidy.^39^ The cyclin-denpendent kinase (CDK) inhibitor p21, which is regulated by p53 at the transcriptional level, has been reported to affect S-phase progression, G2/M arrest, and polyploidization.^26, 40, 41^ As shown in our previous study,^14^ the expression levels of p16^ink4α^, p21, and p53 were higher in octoploid hepatocytes of 18-month age mice, and the levels were lowered with rejuvenation and ploidy reversal.

**Figure 3:**
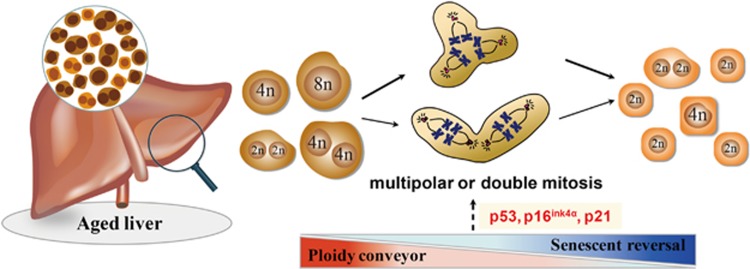
Senescent polyploid hepatocytes are rejuvenated accompanied by ploidy converyor. In the aged liver, senescent polyploid hepatocytes reenter cell cycles after transplantation. These proliferating tetraploid or octoploid hepatocytes can generate mononucleated diploid and tetraploid hepatocytes, as well as binucleated tetraploidy with triploar or double mitosis. Accompanied with senescent hepatocytes reversal, the percentage of octoploid hepatocytes decreases while the percentage of diploid hepatocytes increases. The p16^ink4α^ and p53-p21 pathway regulating hepatocyte senescence may be a mechanism of polyploidization during aging

Consistent with our report, increased polyploidy after two-thirds partial hepatectomy in rats or mice exhibit senescence-type changes with increased β-galactosidase activity and accumulation of p21.^22, 36^ Moreover, with increased senescent polyploidy, the proliferative capacity of partial hepatectomy-induced hepatocytes was significantly attenuated compared to hepatocytes from normal rats.^36^ However, ploidy reversal with multipolar mitosis exists during hepatocyte proliferation.^13^ As shown in our previous study,^14^ repopulated hepatocytes collected from 10-week post-transplantation of 18-month donor cells by flow cytometry and found that the ratio of diploid, tetraploid, and octoploid cells became similar to that seen in the liver of 2-month mice. The percentage of octoploid hepatocytes decreased from 34.06±1.8% before transplantation to 17.4±0.96%, while the percentage of diploid hepatocytes increased from 13.6±0.66% to 21.96±1.78%. Furthermore, isolated octoploid hepatocytes from 18-month-old mice would completely repopulate in young Fah^−/^^−^ recipients at 10 weeks post-transplantation. Analysis of repopulated hepatocytes showed that pure octoploid cells produced daughter cells with 2c and 4c DNA content. And mitotic structures with multipolar spindles or tripolar division were detected during hepatocyte proliferation *in vivo*. In addition, F-actin and Hoechst 33342 co-staining in the repopulated livers indeed revealed the existence of ploidy reversal with reduction in DNA content after proliferation. Accompanied by polyploidy reversal, senescent polyploid hepatocytes became rejuvenated as represented by increased proliferative capacity, decreased β-galactosidase activity and expression of p16^ink4α^, p21, and p53 (Figure 3). According to these data, ploidy reversal with rejuvenation of proliferative capacity may provide a potential clue to develop promising therapeutic strategies for age-dependent diseases.

## Hepatocyte Polyploidization and Liver Dysfunction

Differentiated hepatocytes retain a high proliferative capacity. Upon tissue injury such as partial hepatectomy (PHx), toxin, and drug-induced liver disease, the quiescent hepatocytes will reenter into the cell cycle and contribute to the recovery of the injured liver. Mononucleated and binucleated polyploid hepatocytes accumulated during two-thirds partial hepatectomy-induced liver regeneration.^42^ Although all hepatocytes entered into the cell cycle after 70% partial hepatectomy, only a few continue through the M phase. Hepatic polyploidy can also be modified by metabolic overload, DNA damage, and chemical-induced liver injury. It was reported that in the long-evans cinnamon (LEC) rat, an animal model of Wilson’s Disease, with copper and iron overloads in the liver, hepatocytes had increased polyploidy with delayed mitotic progression.^43^ Furthermore, polyploidy was further increased following exposure to radiation or oxidative stress.^44^ In contrast, hepatocellular carcinoma was shown to have a lower overall polyploidy compared to the normal liver.^45, 46^ Diploid mononucleated hepatocytes increased in frequency with the decrease of polyploidy hepatocytes in both rat models induced by chemical carcinogens (diethylnitrosamine (DEN) and 2-acetyl-aminofluorene (2-AAF)) and human hepatocellular carcinoma (HCC).^45, 46^ Reduced ploidy in HCC may reflect an inverse correlation between growth capacity and ploidy, since it has been reported that HCC’s hepatocytes proliferate as diploid cells.^12^ In addition, diploid cells are less protected against mutagenic change than polyploid cells. Thus, rapidly dividing diploid tumor cells are more easily mutated than the polyploid cells, resulting in increasing malignancy.^47, 48^ HCC development is accompanied by decreased expression of miR-122. MiR-122, frequently the most specific miRNA in the liver, is considered necessary and sufficient for liver polyploidization, in addition to being an important tumor suppressor in hepatocellular carcinoma.^49, 50, 51^ Thus, the data implicate decreased miR-122 level as key to suppressed polyploidization in mouse or rat models of DEN-induced HCC and human HCC. Additional factors, such as survivin, may also play a role. Survivin is overexpressed in human tumor and survivin deficiency induces polyploidy and cell cycle arrest in hepatocellular carcinoma cells,^52^ which suggest deletion of survivin promotes liver polyploidization. Additional studies have demonstrated increased polyploidization in cancer cells treated with mitotic kinase inhibitors or spindle inhibitor nocodazole. Intriguingly, polyploid cancer cells are more sensitive to genotoxic stress and anticancer agents.^53, 54, 55^ Together, these observations suggest that manipulation of polyploidization may be an important strategy to induce cell death in cancer therapies.

**Figure 4:**
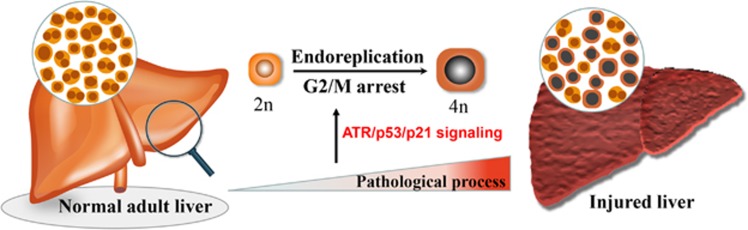
Pathological polyploidization in the liver. In the pathological liver, hepatocytes progress through G1 and enter S-phase, but fail with cell mitosis, leading to formation of mononucleated polyploid hepatocytes. The G2/M arrest is controlled by p53/p21 signaling pathway

Recently, Gentric *et al.* examined hepatic ploidy in a nonalcoholic fatty liver disease (NAFLD) model of ob/ob mice and wild-type (WT) mice fed with methionine-choline-deficient diet (MCD) or high-fat diet (HFD), and found a large proportion of highly polyploid hepatocytes in the parenchyma of the fatty liver.^56^ Most importantly, a similar phenotype was also observed in patients with nonalcoholic steatohepatitis. Hepatocytes of NAFLD in primary cultures progressed through G1 and entered S-phase, but they had delayed exit from S phase and accumulated/arrested in G2, suggesting that endoreplication might be a mechanism for polyploidy (Figure 4). Moreover, the NAFLD hepatocytes in S-phase and G2 displayed a high level of oxidative stress accompanied by robust phosphorylation of ATR (an indicator of DNA damage response) and markers for cell-cycle arrest (phosphorylation of p53 and increased p21 expression). Together, these observations indicate that pathological polyploidization is promoted with oxidative stress by activating the ATR/p53/p21 signaling axis.^56, 57, 58^

## Biological Significance of Hepatocyte Polyploidization in the Liver

The biological significance of liver polyploidization remains enigmatic, but a number of hypotheses have been considered. One hypothesis suggests that hepatic polyploidy is associated with hepatocyte maturity and terminal differentiation.^7, 18, 35, 59, 60^ On the basis of the blood vessel location and blood flow direction, the liver lobule can be subdivided into an upstream ‘periportal’ and a downstream ‘pericentral’ zone. Hepatocytes are believed to mature as they transit, or ‘stream’ from the periportal to pericentral region.^35, 59, 60^ A number of studies have indicated that diploid hepatocytes are located in the periportal region, while downstream, and older pericentral hepatocytes exhibit greater ploidy. However, a more recent study failed to support these observations, and it indicates that diploidy and polyploidy are distributed randomly throughout the hepatic lobule.^17^ Importantly, polyploid hepatocytes are likely not the terminal form of differentiation, since there was no significant difference in regenerative capacity between the liver of E2f8^−/−^ mice with predominant diploid hepatocytes and the adult wild-type liver.^16^ Competitive repopulation of co-transplanted diploid and octoploid hepatocytes isolated from 8-week-old mice into fumarylacetoacetate hydrolase (Fah)-deficient mice shows that the proliferative capacity was similar between the ploidy classes.^13^ In addition, isolated diploid, tetraploid, and octoploid hepatocytes that were separately transplanted all could complete repopulation with similar proliferative capacity.^14^ The data indicate that polyploidization in the young normal liver is not an accurate hallmark for terminal differentiation and has no obvious impact on proliferation in physiological process. However, in the aging or diseased liver, there appears an association of senescence with polyploidization. In our study, we observed that polyploid hepatocytes from old mice had less proliferative capacity and also expressed senescent markers.^14^ During liver regeneration after partial hepatectomy, polyploidy increased with senescence-type changes and the rate of proliferation of polyploid hepatocytes was lower than that of diploid hepatocytes.^36^

A second hypothesis suggests that liver ploidy may serve to enhance hepatocyte function. The liver participates in a wide array of activities related to protein synthesis/secretion, metabolism, and detoxification. Polyploidy may allow two- or four-fold increased expression of some genes/proteins and thereby enhance particular metabolic functions. However, comparison of gene expression profiles of isolated diploids, tetraploids and octoploids by microarray analysis showed that only 50 candidate genes from a wide range of different biological processes were differently expressed.^61^ Whether or not liver polyploidization enhances, hepatocytes function remains debatable. However, a recent study used comparative genome-scale analysis to demonstrate that polyploidy boost tissue-specific functions. Polyploidy-activated genes are present in all essential liver-specific functions, including nitrogenous metabolism, blood protein synthesis, redox state maintance, xenobiotic metabolism, and immunity. Moreover, the study revealed a tendency of the polyploidy liver to an increased anaerobic energy production and obtained ATP from carbohydrates rather than from fatty acids, suggesting polyploidy is associated with the switch of liver-specific functions to economy saving mode, instead of investing it into cell division.^62, 63^

A third hypothesis suggests that polyploidy confers protection of hepatocytes against oxidative stress and genotoxic damage. Acquiring multiple sets of chromosomes could function to buffer against gene-inactivating mutations by DNA damaging agents. For instance, early tumor lesions in the liver are characterized by the increase of diploid cells, which are less protected against mutations than polyploid liver cells.^64^ Notably, genome-scale comparison of diploid and polyploid hepatocytes indicates that polyploidy induces genes fighting against pathogens, DNA lesions and oxidative stress, and inhibits genes promoting apoptosis.^62, 63^ In addition, the progressive polyploidization observed during the liver aging process, implying polyploidization allow the liver to adapt to aging-related cellular loss or may be a protective response to the accumulation of damaged DNA. Under pathological condition, the injury liver with loss of liver function may try to compensate the loss of its mass by polyploidization.

## Conclusion

Polyploid cells, occurring with aging, oxidative stress, and DNA damage, are frequently found in various mammalian organs and tissues, including the skeletal muscle, heart, placenta, brain, liver, and blood cells. In the liver, the first wave of polyploidy is seen at postnatal growth, and the second wave of ploidy elevation is observed at senescence. Many previous studies have indicated that polyploid cells can proliferate as well as diploid hepatocytes in the developmental liver, suggesting that polyploidization is not required for hepatocyte differentiation. Polyploid hepatocytes become senescent and decrease their proliferative capacity with aging and pathological process, but senescent polyploid hepatocytes can rejuvenate with ploidy reversal. The molecular cues controlling polyploidization and reversal remain unknown, and a better understanding of ploidy reversal is needed to approach clinical research. Under pathological conditions, liver polyploidization mainly indicates the severity of the damage: the higher the observed polyploidization rate, the greater the injury that has occurred. However, increase of diploid cells is a main characteristic feature in hepatocellular carcinoma, owing to their heightened proliferative capacity and their susceptibility to further mutations. Induced polyploidization could be an underlying strategy for cancer therapies. Finally, further work is also needed to unravel what diseases are associated with pathological polyploidization, how polyploidy is regulate during pathological progression, and whether these new insights will provide more clues for therapeutic strategies in liver diseases.

## Figures and Tables

**Table 1:** Genes contribute to polyploidy

**Gene symbol**	**Gene function**	**Genetic modification**	**Effect on liver polyploidization**	**References**
*E2f7/8*	Cell cycle transcription factor	Deletion	Impair polyploidization	^16, 24^
*Ccne1*	Cell cycle, G1/S transition	Deletion	Impair polyploidization	^25^
*E2f1/2/3*	Cell cycle transcription factor	Deletion	Promote polyploidization	^23, 24^
*Trp53*	Cell cycle factor, tumor supressor	Deletion	Promote polyploidization	^26, 27^
*p21*	Cell cycle factor	Deletion	Promote polyploidization	^26^
*Rb*	Cell cycle, mitosis	Deletion	Promote polyploidization	^26, 28^
*Cdk1*	Cell cycle, mitosis	Deletion	Promote polyploidization	^29^
*Ccne2*	Cell cycle, G1/S transition	Deletion	Promote polyploidization	^25^
*Skp2*	Cell cycle	Deletion	Promote polyploidization	^30^
*Ssu72*	Cell cycle	Deletion	Promote polyploidization	^31^
*ERCC1*	DNA repair	Deletion	Promote polyploidization	^15^
*Survivin*	Cell cycle, mitosis	Deletion	Promote polyploidization	^32, 33^
*miR-122*	Proliferation	Overexprssion	Promote polyploidization	^34^
